# Supplementary material for: m6A and the NEXT complex direct Xist RNA turnover and X-inactivation dynamics
Source: Nat Struct Mol Biol. 2025 Sep 9;32(11):2242–51. doi: 10.1038/s41594-025-01663-w (PMC12618237; doi:10.1038/s41594-025-01663-w)
Supplement: Supplementary file 1 — Supplementary Figs. 1 and 2 and Tables 1 and 2. [file 41594_2025_1663_MOESM1_ESM.pdf]

nature structural & molecular biology

Supplementary information https://doi.org/10.1038/s41594-025-01663-w

m^{6}A and the NEXT complex direct Xist RNA turnover and X-inactivation dynamics

In the format provided by the authors and unedited

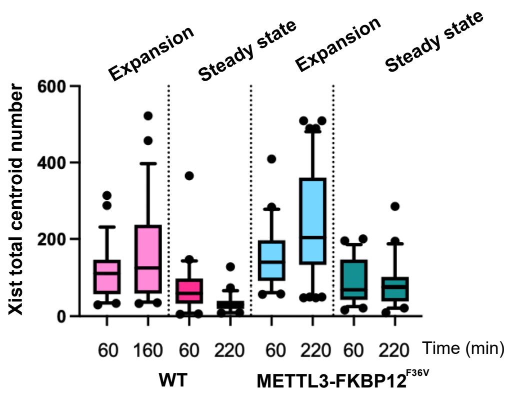

Supplementary Fig. 1: Xist RNA levels are elevated following acute METTL3 depletion.

Boxplots quantifying total Xist centroid numbers, assessed by RNA-SPLIT, in WT and METTL3 dTAG-depleted cells at both expansion and steady-state phases (corresponding to Fig. 3c). Xist centroid levels are measured at the start and end of each time course. Centre line, median; box, 25th and 75th percentiles; whiskers, 1.5× interquartile range.

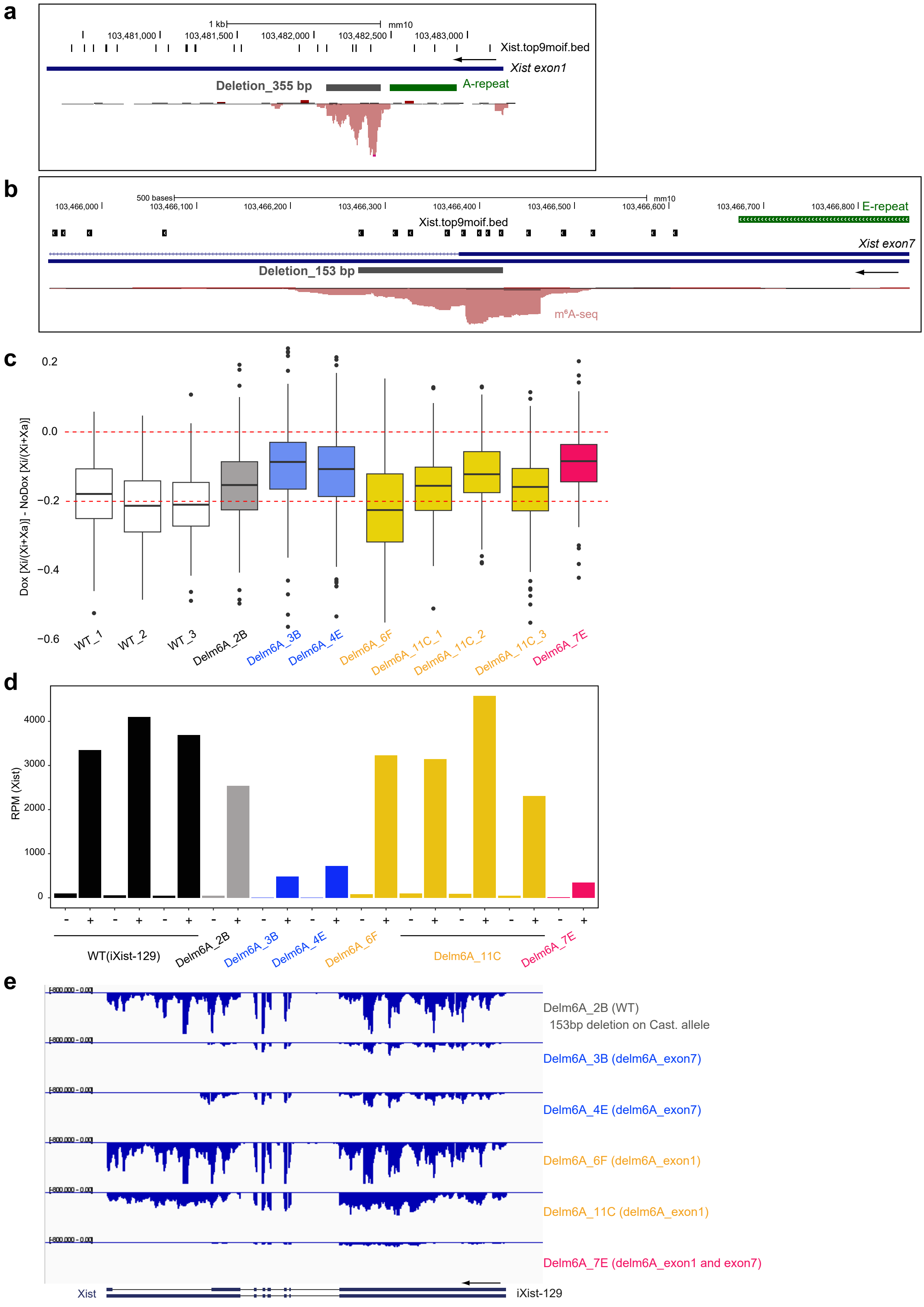

a
1 kb
mm10
103,481,000
103,481,500
103,482,000
103,482,500
103,483,000
Xist.top9moif.bed
Xist exon1
Deletion_355 bp
A-repeat
b
500 bases
mm10
103,466,000
103,466,100
103,466,200
103,466,300
103,466,400
103,466,500
103,466,600
103,466,700
103,466,800
E-repeat
Xist.top9moif.bed
Xist exon7
Deletion_153 bp
m6A-seq
c
Dox [Xi/(Xi+Xa)] - NoDox [Xi/(Xi+Xa)]
0.2
0.0
−0.2
−0.4
−0.6
WT_1
WT_2
WT_3
Delm6A_2B
Delm6A_3B
Delm6A_4E
Delm6A_6F
Delm6A_11C_1
Delm6A_11C_2
Delm6A_11C_3
Delm6A_7E
d
RPM (Xist)
4000
3000
2000
1000
0
WT(iXist-129)
Delm6A_2B
Delm6A_3B
Delm6A_4E
Delm6A_6F
Delm6A_11C
Delm6A_7E
e
Delm6A_2B (WT)
153bp deletion on Cast. allele
Delm6A_3B (delm6A_exon7)
Delm6A_4E (delm6A_exon7)
Delm6A_6F (delm6A_exon1)
Delm6A_11C (delm6A_exon1)
Delm6A_7E (delm6A_exon1 and exon7)
Xist
iXist-129

Supplementary Fig. 2: Analysis of Xist RNA levels and XCI dynamics following deletion of m^{6}A regions of Xist exon 1 and exon 7.

a,b, UCSC genome browser view of m^{6}A regions downstream of Xist A-repeat (**a**) and E-repeat (**b**), alongside the Xist repeat annotation and m^{6}A motifs (DRACH). Xist transcription direction is indicated by the arrowhead.

c, Boxplots illustrating changes in allelic expression ratios of X-linked genes between Dox 24 h and NoDox conditions. Red lines indicate allelic ratios of 0 (no silencing) and –0.2 (representing the typical silencing level in WT after 24 h of Dox induction). Blue boxes correspond to exon 7 m^{6}A region deletions, yellow boxes to exon 1 m^{6}A region deletion, and the purple box to combined exon 1 and exon 7 m^{6}A region deletions. Each biological replicate is represented by a separate box. Center line, median; box, 25th and 75th percentiles; whiskers, 1.5× interquartile range.

d, Barplot showing Xist RNA expression level at the indicated condition. Each bar represents one biological replicate.

e, UCSC genome browser view of Xist expression profile. A summary of deletions is shown to the right. Xist RNA gene structure and transcription direction are shown at the bottom.

Supplementary Table 1: A summary table of cell lines generated in this study.

Degron line	Clone Name	Xist Type	XX karyotype
N-terminal METTL3	Clone1 (G4)	Xist	XX
N-terminal METTL3	Clone2 (H1)	Xist	XX
C-terminal METTL3	Clone1 (C3)	Xist	XX
C-terminal METTL3	Clone2 (H5)	Xist	XX
C-terminal METTL3 degron (H5) + Rosa_WT_METTL3	Clone1 (WT_P2B3)	Xist	XX
C-terminal METTL3 degron (H5) + Rosa_WT_METTL3	Clone2 (WT_1A)	Xist	XiO
C-terminal METTL3 degron (H5) + Rosa_WT_METTL3	Clone3 (WT_10C)	Xist	XiO
C-terminal METTL3 degron (H5) + Rosa_D395A_METTL3	Clone1 (D395A_1F)	Xist	XX
C-terminal METTL3 degron (H5) + Rosa_D395A_METTL3	Clone2 (D395A_5B)	Xist	XiO
C-terminal METTL3 degron (H5) + Rosa_D395A_METTL3	Clone3 (D395A_9F)	Xist	XiO
C-terminal METTL3	Clone3 (M12D)	Xist-BgI	XX
C-terminal YTHDC1	Clone1 (Y9F)	Xist-BgI	XX
C-terminal YTHDC1	Clone2 (Y12H)	Xist-BgI	XX
C-terminal ZCCHC8	Clone1 (Z4F)	Xist-BgI	XX
C-terminal ZCCHC8	Clone2 (Z12H)	Xist-BgI	XX
C-terminal ZCCHC8	Clone3 (Z3A)	Xist-BgI	
C-terminal ZFC3H1	Clone1 (8H)	Xist	XX
C-terminal ZFC3H1	Clone2 (10A)	Xist	
C-terminal ZFC3H1	Clone3 (11A)	Xist	XX [0-138M]
Note: XX (both inactive and active X are present); XiO (only the inactive X is present, but the active X is not detected); XX [0-138M] (The inactive X is SNP informative from 0-138M), empty means not characterised.			

Deletions generated in the background of iXist-ChrX129.

	Exon 1 m6A region	Exon 7 m6A region	note
Xist_Delm6A_2B	Cast (WT);<br>129 (WT)	Cast (153bp deletion);<br>129 (WT)	WT Xist
Xist_Delm6A_3B	Cast (WT);<br>129 (WT)	Cast (153bp deletion);<br>129 (153bp deletion)	study m6A in Exon7
Xist_Delm6A_4E	Cast (WT);<br>129 (WT)	Cast (153bp deletion);<br>129 (153bp deletion)	study m6A in Exon7
Xist_Delm6A_6F	Cast (WT);<br>129 (355bp deletion)	129 (WT)	Study m6A in Exon1
Xist_Delm6A_7E	Cast (WT);<br>129 (355bp deletion)	Cast (WT);<br>129 (153bp deletion)	study m6A in Exon1 and Exon7

Supplementary Table 2: Primers used in this study.

used for amplifying FKBP12^F36V
>FKBP12_F
gtggaaaccatctccccaggag
>FKBP12_R
ttccagttttagaagctccacatcgaagac

Gibson Cloning primers used for Ythdc1 C-term homology arm and FKBP12^F36V knockin
>Ythdc1C_Frag1F
GGGTTCCGCGCACATTTCCCCGAAAAGTGCCACCTGACGTGAAATTACAGGAATACATGGTGCT
CTAGTTAGT
>Ythdc1C_Frag1R
ccatGCCTCCACTTCCACCTCTTCGATAACGACCTCTCTCCCCTCATTCTCTGTCCC
>Ythdc1C_Frag2F
AGAGAGGTCGTTATCGAAGAGGTGGAAGTGGAGGCatgg
>Ythdc1C_Frag2R
GTGCTTCCAAAAGCACATTAttccagttttagaagctccacatcgaagac
>Ythdc1C_Frag3F
tggagcttctaaaactggaaTAATGTGCTTTTGGAAGCACTGACT
>Ythdc1C_Frag3R
TATCGATGCGGCCGCGCTAGCACGCGTCAGCTGACTAGAGGAAATTCTATGGCAGTTATTGACC
AAGGTCC
>Ythdc1_dTAG_ATTA_R
cgacctctctcccctctatctctgtcccgatcac
>Ythdc1_dTAG_ATTA_F
gtgatcgggacagagatagaggggagagaggtcg

used for knockin validation
>Ythdc1_CF
GGGATGGAGTGAGTGAACAAG
>Ythdc1_CR
ACTGAACCGTGAGCAGCTAC

used for sgRNA cloning
>Ythdc1C_sgRNA_F
caccGATAACGACCTCTCTCCCCT
>Ythdc1C_sgRNA_R
aaacAGGGGAGAGAGGTCGTTATC

Gibson Cloning primers used for Zcchc8 C-term homology arm and FKBP12^F36V knockin
>Zcchc8C_Frag1F
GGGTTCCGCGCACATTTCCCCGAAAAGTGCCACCTGACGTGTGTCCAAACGAGTTTGACCTCC
>Zcchc8C_Frag1R
cccatGCCTCCACTTCCACCCTCACAAGTCTTTTTGTTTTTCTGCTGATTTCG
>Zcchc8C_Frag2F
AAAACAAAAAGACTTGTGAGGGTGGAAGTGGAGGCatgg
>Zcchc8C_Frag2R
CACATCAATACGCAGCCAGGGCTCAttccagttttagaagctccacatc
>Zcchc8C_Frag3F
gagcttctaaaactggaaTGAGCCCTGGCTGCGTATTGATGTGCAGCTCTGA
>Zcchc8C_Frag3R
TATCGATGCGGCCGCGCTAGCACGCGTCAGCTGACTAGAGAATACCCCGAGCAAGCAGG

used for knockin validation
>Zcchc8_CF
GAGGGAATGGCGACTCAGATG
>Zcchc8_CR
TGTACACAAAGGTGAGCACACC

used for sgRNA cloning
>Zcchc8C_sgRNA_F
caccGTGAGTGAGCCCTGGCTGCG
>Zcchc8C_sgRNA_R
aaacCGCAGCCAGGGCTCACTCAC

used for sgRNA cloning
>Mettl3N_sgRNA_F
caccgCCTAGCTCCCAGCGCGGACT
>Mettl3N_sgRNA_R
aaacAGTCCGCGCTGGGAGCTAGGc

Gibson Cloning primers used for Mettl3 N-term homology arm and FKBP12^F36V knockin
>Mettl3N_Frag1F
CACATTTCCCCGAAAAGTGCCACCTGACGTtctatgatacagtgcttgaccgtatgtgc
>Mettl3N_Frag1R
ggtttccacctgcactcccatCTCGAATCAGGCGCGGCGGA
>Mettl3N_Frag2F
TCCGCCGCGCCTGATTCGAGatgggagtgcaggtggaaacc
>Mettl3N_Frag2R
CCACGTGTCCGACATGCCTCCACTTCCACCt
>Mettl3N_Frag3F
GGTGGAAGTGGAGGCATGTCGGACACGTGGAGC
>Mettl3N_Frag3R
GCCGCGCTAGCACGCGTCAGCTGACTAGAGtctcggaataaggtactgagaggcttcacg
>Insert_sgRNA_F
cgcgcctgattcgagaggagtccgcgctgggagctaggatgggagtgcaggtg
>Insert_sgRNA_R
cacctgcactcccatcctagctcccagcgcggactcctctcgaatcaggcgcg

used for knockin validation
>Mettl3_NF
gaaaatctggggccttaagggc
>Mettl3_NR
gctaaaccgggctctggga

>GW107_Mettl3_CF
AATTGACGTGGACTGGGCAT
>GW108_Mettl3_CR
ATTGGGCTAGAGGGGAAACGA

used for LIC cloning METTL3 to plasmid#669 that has N-teminal GFP
>Mettl3_LIC_F
TACTTCCAATCCATGTCGGACACGTGGAGCTCT
>Mettl3_LIC_R
TATCCACCTTTACTGTCTATAAATTCTTAGGTTTAGAGATGATG

used for GFP-METTL3 expression from Rosa26 locus
>GFP-METTL3_F
cggtctttccagtggggatcgacggtatcgaattcacccaatggtgagcaagggcgaggag
>GFP-METTL3_R
TGTAACCATTATAAGCTGCAATAAACAAGTTAACAACAACCTATAAATTCTTAGGTTTAGAGATGAT
GCCGTCCG
>D395A_R
tgtgaatatcccaaggtggtgcagccatcacaactgcaaa
>D395A_F
tttgcagttgtgatggctgcaccaccttgggatattcaca

primers used for ZFC3H1 dTAG degron
used for gDNA insertion validation
>GW246_ZFC3H1C_gDNA_F
GCCATTGCTGCTGAGATTGC
>GW247_ZFC3H1C_gDNA_R
CAACTAGCAGACTTGTGGCCG

used for Gibson Assembly
>GW248_ZFC3H1C_Frag1F
GGGTTCCGCGCACATTTCCCCGAAAAGTGCCACCTGACGTAGCCATGTTTGACTTGAGTTCTAG
TG
>GW249_ZFC3H1C_Frag1R
cccatGCCTCCACTTCCACCGAGATTCTTGCTTTCTGTTTTGTTACTGTTTAAATTTAAGAG
>GW250_ZFC3H1C_Frag2F
AAACAGAAAGCAAGAATCTCGGTGGAAGTGGAGGCatg
>GW251_ZFC3H1C_Frag2R
CTGACTGCACCCAGTGTTCAttccagttttagaagctccacatcg
>GW252_ZFC3H1C_Frag3F
tggagcttctaaaactggaaTGAACACTGGGTGCAGTCAGT
>GW253_ZFC3H1C_Frag3R
TATCGATGCGGCCGCGCTAGCACGCGTCAGCTGACTAGAGGACTGCCTGCTATTTTACTGTAAA
C

used for ZFC3H1 C-termal sgRNA cloning
>GW254_ZFC3H1C_sgRNA_F
caccgAAGCAAGAATCTCTGAACAC
>GW255_ZFC3H1C_sgRNA_R
aaacGTGTTCAGAGATTCTTGCTTc

sgRNA cloning used for Xist exon7 m6A region deletion
>XistExon7_sgRNA_F
caccgCCTAAGGACCCAATCCTATA
>XistExon7_sgRNA_R
aaacTATAGGATTGGGTCCTTAGGc

used for deletion validation (WT:358bp)
>XistExon7_Deletion_F
GGTCAGGATTCAAGTGGCTC
>XistExon7_Deletion_R
GGGCAGGTCTCATCTTCGAA
