# Supplementary material for: m6A and the NEXT complex direct Xist RNA turnover and X-inactivation dynamics
Source: Nat Struct Mol Biol. 2025 Sep 9;32(11):2242–51. doi: 10.1038/s41594-025-01663-w (PMC12618237; doi:10.1038/s41594-025-01663-w)
Supplement: Supplementary file 2 — Reporting Summary [file 41594_2025_1663_MOESM2_ESM.pdf]

nature portfolio

Corresponding author(s): Guifeng Wei and Neil Brockdorff

Last updated by author(s): Jun 17, 2025

Reporting Summary

Nature Portfolio wishes to improve the reproducibility of the work that we publish. This form provides structure for consistency and transparency in reporting. For further information on Nature Portfolio policies, see our Editorial Policies and the Editorial Policy Checklist.

Statistics

For all statistical analyses, confirm that the following items are present in the figure legend, table legend, main text, or Methods section.

n/a	Confirmed	
☐	☒	The exact sample size (*n*) for each experimental group/condition, given as a discrete number and unit of measurement
☐	☒	A statement on whether measurements were taken from distinct samples or whether the same sample was measured repeatedly
☐	☒	The statistical test(s) used AND whether they are one- or two-sided<br>*Only common tests should be described solely by name; describe more complex techniques in the Methods section.*
☒	☐	A description of all covariates tested
☒	☐	A description of any assumptions or corrections, such as tests of normality and adjustment for multiple comparisons
☐	☒	A full description of the statistical parameters including central tendency (e.g. means) or other basic estimates (e.g. regression coefficient) AND variation (e.g. standard deviation) or associated estimates of uncertainty (e.g. confidence intervals)
☐	☒	For null hypothesis testing, the test statistic (e.g. *F*, *t*, *r*) with confidence intervals, effect sizes, degrees of freedom and *P* value noted<br>*Give P values as exact values whenever suitable.*
☒	☐	For Bayesian analysis, information on the choice of priors and Markov chain Monte Carlo settings
☒	☐	For hierarchical and complex designs, identification of the appropriate level for tests and full reporting of outcomes
☒	☐	Estimates of effect sizes (e.g. Cohen's *d*, Pearson's *r*), indicating how they were calculated

Our web collection on statistics for biologists contains articles on many of the points above.

Software and code

Policy information about availability of computer code

Data collection	Sequencing data for ChrRNA-seq, SLAM-seq, MeRIP-seq, and total RNA-seq were generated using the Illumina NextSeq 500 platform. RNA-SPLIT images were acquired with the DeltaVision OMX V3 Blaze system (GE Healthcare). Western blot images were developed using a Konica SRX-101A Medical Film Processor and scanned with a TASKalfa 5004i. qPCR was performed on the Rotor-Gene Q (QIAGEN).
Data analysis	Bowtie2 (2.3.5 & 2.4.5), SAMtools (1.16.1), STAR (v2.5.2b & 2.7.9a), SNPsplit (0.4.0dev), IGV (2.17.1), Subread (1.5.2), Picard tools (2.25.0), deeptools (3.5.5), bedtools (v2.27.1), Python (3.8.10), TEtranscripts (v2.2.1), R (4.1.0 & 4.2.1), and tidyverse (2.0.0). RNA-SPLIT analysis code has been deposited in github (https://github.com/HollyRoach/Automated_RNA-SPLIT).

For manuscripts utilizing custom algorithms or software that are central to the research but not yet described in published literature, software must be made available to editors and reviewers. We strongly encourage code deposition in a community repository (e.g. GitHub). See the Nature Portfolio guidelines for submitting code & software for further information.

Data

Policy information about availability of data

All manuscripts must include a data availability statement. This statement should provide the following information, where applicable:

- Accession codes, unique identifiers, or web links for publicly available datasets
- A description of any restrictions on data availability
- For clinical datasets or third party data, please ensure that the statement adheres to our policy

High-throughput raw sequencing data as well as key processed data, including ChrRNA-seq, SLAM-seq, MeRIP-seq, and total RNA-seq, are deposited to the National Center for Biotechnology Information's Gene Expression Omnibus (accession number GSE279269).
The mouse genome (mm10) sequence and gene annotation were downloaded from UCSC genome browser (https://hgdownload.soe.ucsc.edu/downloads.html).
The whole genome collections of SNP and short indel variants for mouse strains 129S1 and Cast/EiJ (mpg.v5) was downloaded from mouse genome project (https://www.sanger.ac.uk/data/mouse-genomes-project/). Gene categories including initial X-linked gene expression level were taken from GSE119602. Gene silencing kinetics data were taken from GSE185843. Promoter chromatin landscape of mm10 genome were retrieved from (https://github.com/guifengwei/ChromHMM_mESC_mm10).
Uncropped western blots and numerical source data are available in source data. Previously published sequencing dataset and imaging dataset used in this study have been specified in the manuscript.

Research involving human participants, their data, or biological material

Policy information about studies with human participants or human data. See also policy information about sex, gender (identity/presentation), and sexual orientation and race, ethnicity and racism.

Reporting on sex and gender	NA
Reporting on race, ethnicity, or other socially relevant groupings	NA
Population characteristics	NA
Recruitment	NA
Ethics oversight	NA

Note that full information on the approval of the study protocol must also be provided in the manuscript.

Field-specific reporting

Please select the one below that is the best fit for your research. If you are not sure, read the appropriate sections before making your selection.

☒ Life sciences ☐ Behavioural & social sciences ☐ Ecological, evolutionary & environmental sciences

For a reference copy of the document with all sections, see nature.com/documents/nr-reporting-summary-flat.pdf

Life sciences study design

All studies must disclose on these points even when the disclosure is negative.

Sample size	No statistical methods were used to predetermine sample size for RNA-SPLIT and sequencing analysis.<br>For RNA-SPLIT, in theory, analyzing more cells leads to more accurate estimates. A minimum of 20 randomly selected cells at each time point was arbitrarily chosen for the analysis.<br>For western blots, at least two biologically independent replicates were performed.<br>For RNA-seq, either multiple independent clones or 2-3 independent repeats for a single clone were chosen, according to common practice in the field. This design ensures the results are reproducible.
Data exclusions	We confirm that no data were excluded for the analysis.
Replication	The number of biological replicates are indicated in the text, figure legend, or method section.
Randomization	For tissue culture based experiments, all wells in each biological replicate were split from the same batch of cells and randomly divided for each condition.
Blinding	For all the experiments and outcome assessments, the investigators were not blinded. This is because no subjective scoring was pre-required for this study as data analysis was all performed by software programs/algorithms.

Reporting for specific materials, systems and methods

We require information from authors about some types of materials, experimental systems and methods used in many studies. Here, indicate whether each material, system or method listed is relevant to your study. If you are not sure if a list item applies to your research, read the appropriate section before selecting a response.

Materials & experimental systems

n/a	Involved in the study
☐	☒ Antibodies
☐	☒ Eukaryotic cell lines
☒	☐ Palaeontology and archaeology
☒	☐ Animals and other organisms
☒	☐ Clinical data
☒	☐ Dual use research of concern
☒	☐ Plants

Methods

n/a	Involved in the study
☒	☐ ChIP-seq
☒	☐ Flow cytometry
☒	☐ MRI-based neuroimaging

Antibodies

Antibodies used

Primary antibody:
anti-METTL3 (Abcam, ab195352, 1:1000), anti-METTL14 (Sigma-Aldrich, HPA038002, 1:1000), anti-RBM15 (Proteintech, 10587-1-AP, 1:1000), anti-YTHDC1 (Sigma-Aldrich, HPA036462, 1:1000), anti-WTAP (Proteintech, 10200-1-AP, 1:1000), anti-m6A (Synaptic Systems, 202 003), anti-TBP (Abcam, ab51841, 1:1000), anti-SETDB1 (Proteintech, 11231-1-AP, 1:1000), anti-ZCCHC8 (Proteintech, 23374-1-AP, 1:1000), anti-ZFC3H1 (Sigma-Aldrich, HPA007151, 1:1000), and anti-KAP1 (Abcam, ab10484, 1:1000).

Secondary antibody:
anti-rabbit IgG HRP Donkey (Amersham, NA934V, 1:2000) and anti-mouse IgG HRP Sheep (Amersham, NXA931V, 1:2000).

Validation

Antibodies are verified by manufacturers using knockouts according to their websites.
Antibodies against mouse METTL3, YTHDC1, ZCCHC8, ZFC3H1 are also verified in this study because the FKBP-V insertion causes the upshift of the protein and the fusion proteins are sensitive to dTAG-13 treatment.
Antibodies against WTAP and RBM15 are also verified in this study by gene knockout.

anti-METTL3 (https://www.abcam.com/en-us/products/primary-antibodies/mettl3-antibody-epr18810-ab195352)
anti-METTL14 (https://www.sigmaaldrich.com/GB/en/product/sigma/hpa038002)
anti-RBM15 (https://www.ptglab.com/products/RBM15-Antibody-10587-1-AP.htm)
anti-YTHDC1 (https://www.sigmaaldrich.com/GB/en/product/sigma/hpa036462)
anti-WTAP (https://www.ptglab.com/products/WTAP-Antibody-10200-1-AP.htm)
Anti-m6A (https://sysy.com/product/202003)
anti-TBP (https://www.abcam.com/en-us/products/primary-antibodies/tata-binding-protein-tbp-antibody-mabcam51841-bsa-and-azide-free-ab282715)
anti-SETDB1 (https://www.ptglab.com/products/SETDB1-Antibody-11231-1-AP.htm)
anti-ZCCHC8 (https://www.ptglab.com/products/ZCCHC8-Antibody-23374-1-AP.htm)
anti-ZFC3H1 (https://www.sigmaaldrich.com/GB/en/product/sigma/hpa007151)
anti-KAP1 (https://www.abcam.com/en-us/products/primary-antibodies/kap1-antibody-ab10484)
anti-rabbit IgG HRP Donkey (https://www.cytivalifesciences.com/en/us/shop/protein-analysis/blotting-and-detection/blotting-standards-and-reagents/amersham-ecl-hrp-conjugated-antibodies-p-06260).
anti-mouse IgG HRP Sheep (https://www.cytivalifesciences.com/en/us/shop/protein-analysis/blotting-and-detection/blotting-standards-and-reagents/amersham-ecl-hrp-conjugated-antibodies-p-06260).

Eukaryotic cell lines

Policy information about cell lines and Sex and Gender in Research

Cell line source(s)

All mouse embryonic stem cells (mESCs) used in this study are female, derived from the F1 2–1 XX mESC line (129/Sv-Cast/Ei), a gift from by J. Gribnau.

Using this background, we developed dox-inducible endogenous Xist cell lines targeted to either the 129S allele (iXist-ChrX129) or the Cast allele (iXist-ChrXCast). For the RNA-SPLIT experiments, we introduced a bgl stem-loop into Xist exon 7 (iXist-bgl) within the iXist-ChrX129 background.

We created N-terminal or C-terminal FKBP-V tagged METTL3 lines on the iXist-ChrXCast background, and C-terminal FKBP-V tagged METTL3 on iXist-bgl-ChrX129. We also generated C-terminal FKBP-V tagged YTHDC1 and ZCCHC8 were established in the iXist-bgl-ChrX129 line, and C-terminal FKBP-V tagged ZFC3H1 in iXist-ChrXCast. To further analyse METTL3 catalytic function, GFP-METTL3 and GFP-METTL3D395A lines were introduced as transgenes at the Rosa26 locus with expression driven by the Rosa26 promoter in one of the C-terminal FKBP-V tagged METTL3 lines in iXist-ChrXCast.

We generated Xist exon 7 m6A region deletions either alone or in combination with Xist exon 1 m6A region deletion in the

	iXist-ChrX129 background.<br><br>The karyotype of the X chromosome in each cell line was confirmed by PCR, as documented in the manuscript.
Authentication	All engineered cell lines were validated at both the genomic level, using PCR, and at the protein level, using Western blot analysis. GFP-METTL3 transgene lines were further validated through microscopy to assess localisation.
Mycoplasma contamination	All the cell lines are regularly tested for mycoplasma contamination, and confirmed to be negative.
Commonly misidentified lines<br>(See ICLAC register)	No commonly misidentified lines were used in this work.

Plants

Seed stocks	Plants are not used in this study.
Novel plant genotypes	Plants are not used in this study.
Authentication	Plants are not used in this study.
