# Supplementary figures and images for: m6A and the NEXT complex direct Xist RNA turnover and X-inactivation dynamics
Source: Nat Struct Mol Biol. 2025 Sep 9;32(11):2242–51. doi: 10.1038/s41594-025-01663-w (PMC12618237; doi:10.1038/s41594-025-01663-w)

Uncropped Western Blot

Fig. 1b

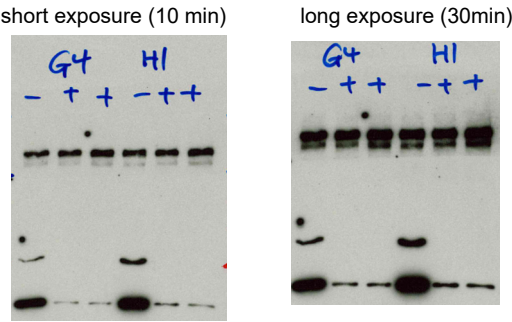

Supplement: Supplementary file 6 — Unprocessed western blots. [file 41594_2025_1663_MOESM6_ESM.pdf]

Uncropped Western Blot

Fig. 4b

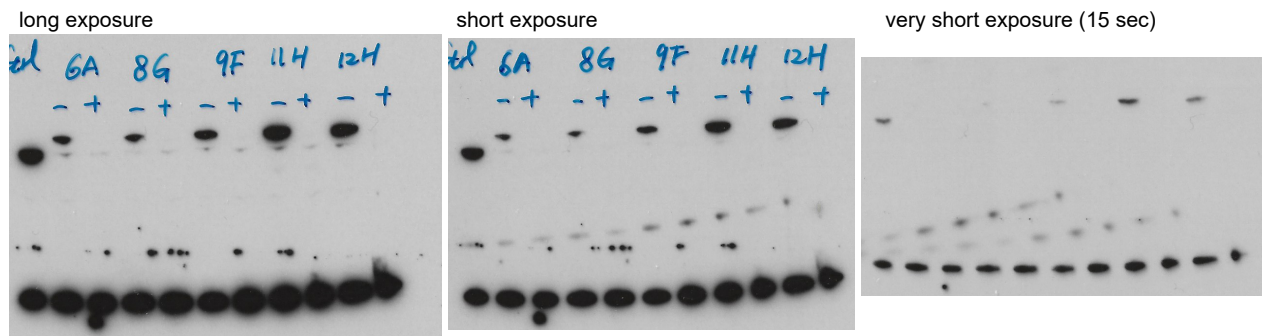

Fig. 4c left

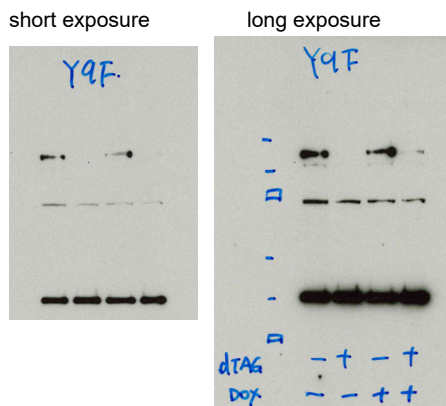

Fig. 4c right

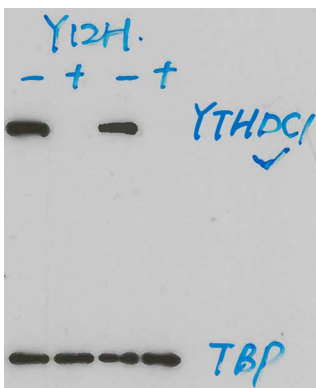

Supplement: Supplementary file 10 — Unprocessed western blots. [file 41594_2025_1663_MOESM10_ESM.pdf]

**Uncropped Western Blot**

**Extended Data Fig. 4a**

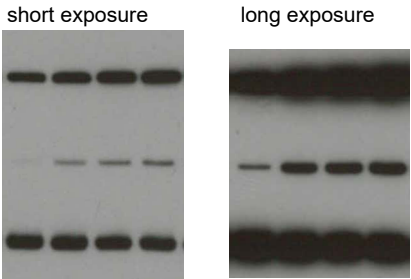

Supplement: Supplementary file 20 — Unprocessed western blots. [file 41594_2025_1663_MOESM20_ESM.pdf]

Uncropped Western Blot

Extended Data Fig. 9b

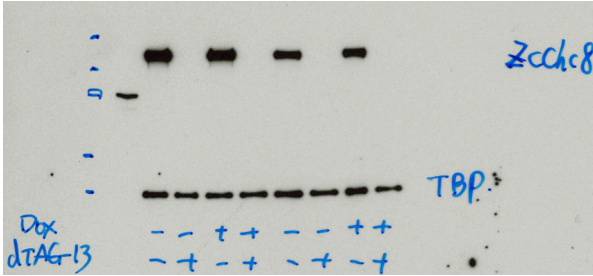

Supplement: Supplementary file 24 — Unprocessed western blots. [file 41594_2025_1663_MOESM24_ESM.pdf]
