# Supplementary material for: m6A and the NEXT complex direct Xist RNA turnover and X-inactivation dynamics
Source: Nat Struct Mol Biol. 2025 Sep 9;32(11):2242–51. doi: 10.1038/s41594-025-01663-w (PMC12618237; doi:10.1038/s41594-025-01663-w)

Uncropped Western Blot

Fig. 5a

ZCCHC8-dTAG

long exposure

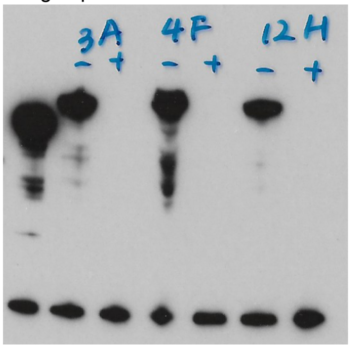

short exposure

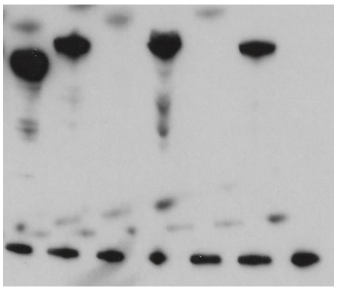

Fig. 5b

ZFC3H1-dTAG

long exposure (30min)

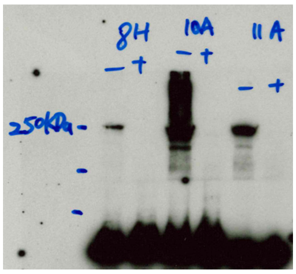

short exposure (1 min)

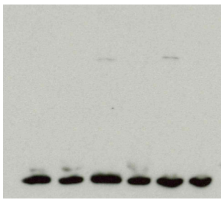

Supplement: Supplementary file 12 — Unprocessed western blots. [file 41594_2025_1663_MOESM12_ESM.pdf]
