# Supplementary material for: m6A and the NEXT complex direct Xist RNA turnover and X-inactivation dynamics
Source: Nat Struct Mol Biol. 2025 Sep 9;32(11):2242–51. doi: 10.1038/s41594-025-01663-w (PMC12618237; doi:10.1038/s41594-025-01663-w)

Uncropped Western Blot

Extended Data Fig. 2a top

long exposure (whole)

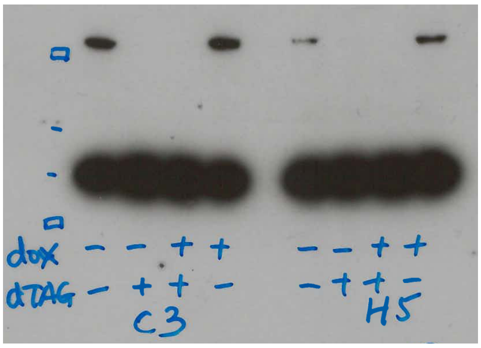

long exposure (only right 4 samples)

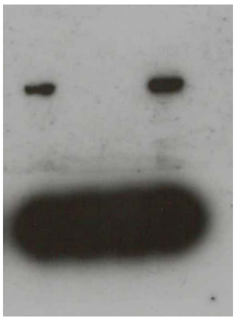

short exposure (10 sec)

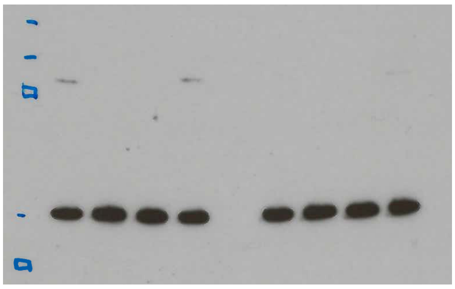

Extended Data Fig. 2a bottom

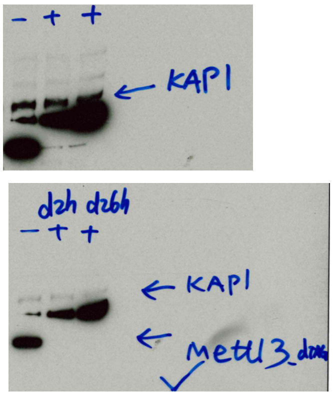

Extended Data Fig. 2b

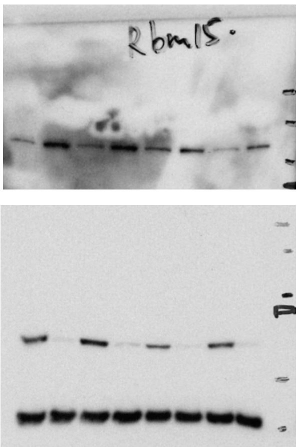

Extended Data Fig. 2c

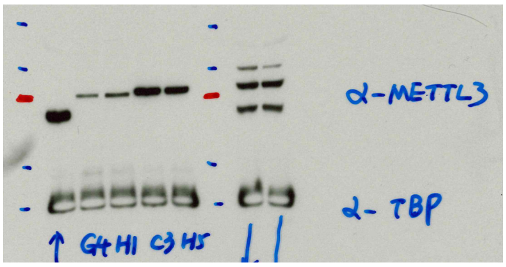

Supplement: Supplementary file 16 — Unprocessed western blots. [file 41594_2025_1663_MOESM16_ESM.pdf]
