# Supplementary material for: m6A and the NEXT complex direct Xist RNA turnover and X-inactivation dynamics
Source: Nat Struct Mol Biol. 2025 Sep 9;32(11):2242–51. doi: 10.1038/s41594-025-01663-w (PMC12618237; doi:10.1038/s41594-025-01663-w)

Uncropped Western Blot

Extended Data Fig. 3b left

long exposure

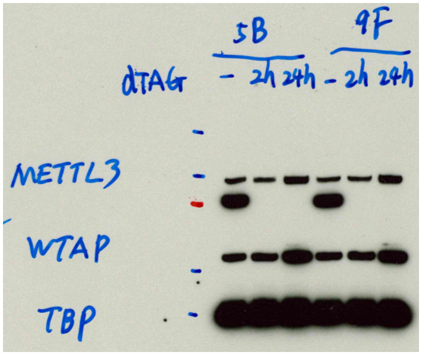

short exposure

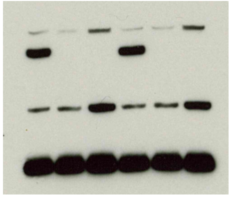

very short exposure (2 sec)

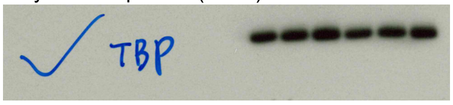

Extended Data Fig. 3b right

short exposure

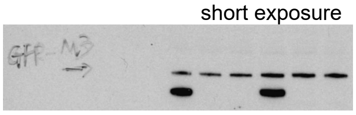

long exposure

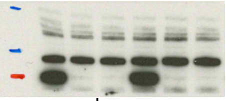

very long exposure

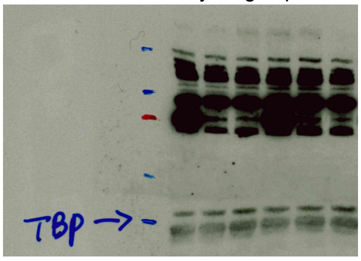

Extended Data Fig. 3b bottome for both left and right

short exposure

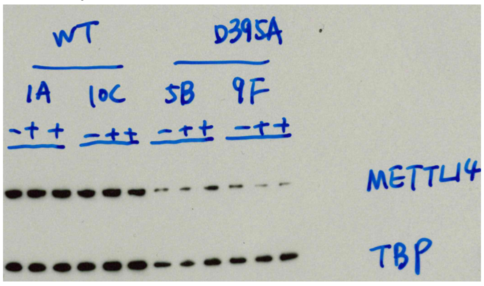

long exposure

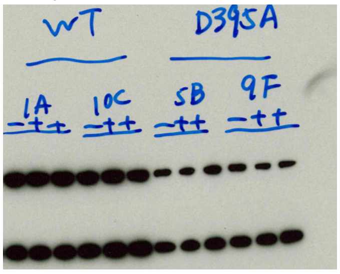

Extended Data Fig. 3c

very short exposure (2 sec)

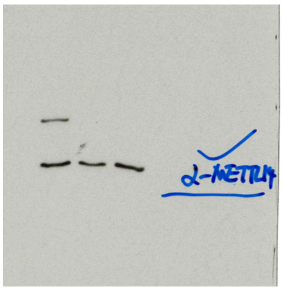

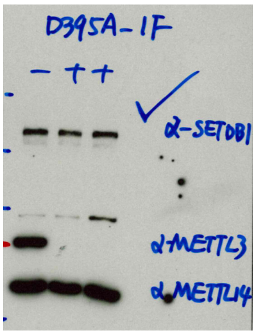

long exposure

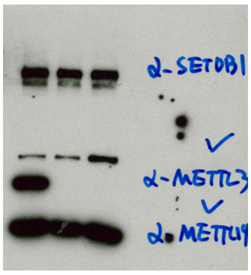

Supplement: Supplementary file 17 — Unprocessed western blots. [file 41594_2025_1663_MOESM17_ESM.pdf]
